# Progress, impacts and lessons from market shaping in the past decade: a systematic review

**DOI:** 10.3389/fpubh.2025.1614471

**Published:** 2025-08-21

**Authors:** Wenhui Mao, Katharine Olson, Elina Urli Hodges, Krishna Udayakumar

**Affiliations:** Duke Global Health Innovation Center, Duke University, Durham, NC, United States

**Keywords:** market shaping, market dynamics, pooled procurement, advance market commitment, product development partnership, demand forecasting, demand generation, systematic review

## Abstract

Market shaping activities have been increasingly used to improve access to health products, such as the advance market commitments used to increase access to the pneumococcal vaccine and COVID-19 vaccines. This paper reviewed the progress and impacts, and identified enablers and barriers of market shaping activities in the past decade. We conducted a systematic review using a structured searching strategy across five academic databases and key actors' websites for gray and white literature published in English since 2012. Two researchers independently performed screening, data extraction, and analysis. Following independent screening, 97 out of 3,006 articles were eligible for analysis. The majority of the articles were qualitative studies and published within the past 5 years. Rapid access to new products, improved availability, and reduced product cost were the most reported impacts. Barriers of market shaping were the disconnection between market shaping interventions and downstream factors, fragmentation and lack of transparency in regulatory processes, and failure to incentivize manufacturers. Enablers included taking end-to-end approaches, coordination across different actors, particularly the national stakeholders and private sector, creating transparent and predictable demand, longer time span, and flexible funding. While market shaping interventions have contributed to the improvement of access to health products, future research should generate additional quantitative evidence, comprehensive impact evaluation, and in-depth studies on the negative impacts of market shaping. Market shaping actors need to adopt definitions and frameworks, apply an ecosystem-wide lens, engage with diverse stakeholders, consider service delivery, and strengthen key capabilities.

**Systematic review registration:**

PROSPERO: CRD42023471098, https://www.crd.york.ac.uk/PROSPERO/view/CRD42023471098

## 1 Introduction

Market dynamics impact the ability of people to receive high-quality, low-cost health products and services, which ultimately affect public health outcomes. All factors involved in the supply and demand for products (the market), including manufacturing, distribution, regulatory, and provider and patient awareness, play a role in determining these outcomes. In many low- and middle-income countries (LMICs), markets are insufficient and inefficient. Consequently, access to effective health products, such as vaccines, drugs, diagnostics, and devices, may be out of reach of those who need them most (1). Various market shaping interventions have been implemented to address market failures. Market shaping refers to using an intervention to address a market failure or shortcoming that compromises health outcomes such as inhibiting access to desired or essential health products and services. Several types of interventions are encompassed by the term market shaping, one example is advanced market commitments (AMCs). In an AMC, donors commit funds to guarantee the price of a health product once it is developed to provide an incentive to manufacturers to invest in research and expansion of capacity for access in LMICs. Volume guarantees are another common type of market shaping intervention where a guarantor enters into an agreement with a manufacturer and agrees to purchase a set quantity of a health product over a period of time, and in return the manufacturer lowers the price. These interventions have been used across the health sector, and across product classes, to bring new suppliers to market, lower the cost of health products, and pool demand and procurement. For example, advanced market commitments, pooled procurement, and other market shaping interventions have been deployed to accelerate access to COVID-19 commodities globally. However, this unprecedented effort has had mixed outcomes, with many of the commodities remaining inaccessible or unaffordable for many low-and-middle income countries (2). For instance, when GeneXpert (a diagnostic platform for infectious diseases) came to market, the cost was much higher than conventional testing approaches (3). Meanwhile, LMICs could not finance a switch to a new testing algorithm and modality, and with uncertain demand, manufacturers were unable to help with scale-up (3). These market failures were addressed when Unitaid and other partners negotiated an upfront payment to lower the price without waiting for sufficient volumes to lead to a price decrease (3).

While there is no consistent definition or a single widely used framework in market shaping yet, several features of market shaping have been summarized to provide a broad picture.

USAID defines market shaping as the act of intervening in a health product market to address market shortcomings such as demand and supply imbalances, and high transaction costs in low-and-middle-income countries (1). Linksbridge's *Foundations of Market Shaping*, describes that market shaping interventions are needed when a market shortcoming or distortion compromises health outcomes such as when market dynamics between buyers and suppliers inhibits access to desired or essential health products and services (4). Some examples of this include information asymmetry between buyers and suppliers, a lack of innovation drivers, and unequal resource distribution (4). Health product markets can fall short across different characteristics, such as information asymmetry between buyers and suppliers or a lack of innovation drivers, and market shaping interventions can span the product lifecycle (1, 4). While there is growing interest in and applications of market shaping, there is limited evidence on its impact. There is even less evidence to identify shared lessons and learnings across different products and health concerns, and from different local contexts. This paper aims to identify trends and patterns of market shaping in LMICs, assess the impacts of market shaping, and better understand the lessons, enablers, and barriers of market shaping in the past decade to inform future market shaping efforts.

## 2 Materials and methods

Review protocol has been registered on PROSPERO (CRD42023471098).

### 2.1 Scope and search strategy

There is no consensus on the definition or framework of market shaping. As described in the introduction section, we used the USAID definition as our working definition of market shaping for this study (11). Our primary focus of this study are market shaping interventions led by global or regional actors that benefit multiple countries. We acknowledge that there are market shaping interventions that have been applied at the national or subnational levels, such as pooled procurement mechanisms led by national governments, but these are not currently considered in our study. To support our research aims, we have three specific objectives for this review: objective 1—identify trends and patterns of market shaping in LMICs; objective 2—assess impacts of market shaping; and objective 3—understand lessons, enablers and barriers of market shaping.

Initial systematic search was conducted in May 2023, for both journal articles and gray literature, with an updated search performed in May 2024. The search included articles published between 2012 and 2024, a period when the majority of market shaping activities have occurred. We limited our search to English language. Additionally, we performed a cross check on the references of included articles, and we obtained articles through internal references.

For journal articles, we performed searches in PubMed, Cochrane, EMBASE, Scopus, and Global Health databases. Databases were decided with input from a librarian specialized in global health. Our search strategy used a series of key terms clustered around three areas in title/abstract/keywords: (1) market shaping (Table 1 provides examples and definitions of specific interventions that are fall under the field of market shaping), (2) health products, and (3) progress. Search string was created using Boolean operators, such that all terms within a given area are connected by an “OR” operator, and areas are connected to one another with an “AND” operator (full searching strategy and examples in Supplementary material S1). We performed a pilot search in PubMed to refine our searching string.

**Table 1:** Examples of different market shaping interventions (5).

**Market shaping interventions**	**Definitions**
R&D; Target Product Profile Issuance	Convene stakeholders to define and publish list of desired product characteristics, use cases, target populations, etc.
R&D; New Product Development	Develop new products to meet TPP, serve new populations, or satisfy other context-specific conditions (i.e., pediatric formulations, fixed-dose combinations, etc.)
R&D; Product Redesign	Improve design of existing products for settings (i.e., improve durability, address infrastructure gaps, etc.)
Regulatory and Normative; Guidelines Inclusion	Support processes for inclusion of new products in guidelines, formularies, and EMLs (i.e., conduct health technology assessments, facilitate guideline review process, etc.)
Manufacture and Commercialization; Licensing Agreements	Enable additional manufacturers to produce and sell on-patent products within a defined territory through voluntary licenses
Manufacture and Commercialization; Strategic Sourcing	Improve sourcing of high-quality active pharmaceutical ingredients, raw materials, and component parts through bulk, direct, and/or local purchasing to reduce overall product cost
Manufacture and Commercialization; Manufacturing Optimization	Identify opportunities to optimize product manufacturing, including via process chemistry, factory automation, packaging redesign, etc.
Manufacture and Commercialization; New Supplier Entry	Support entry of additional suppliers within existing product class to increase total production capacity, diversify supplier base, exert downward pricing pressure, etc.
Manufacture and Commercialization; Commercialization Partnerships	Facilitate agreement of new commercialization partnerships to introduce products (via links between manufacturers, distribution partners, in-country service providers, etc.)
Manufacture and Commercialization; Demand Forecasting	Aggregate on-the-ground data and insights to understand market size and price sensitivity of demand to support supplier negotiations and commercial planning
Manufacture and Commercialization; Price Analysis and Negotiation	Conduct cost of goods sold (COGS), cost-effectiveness, and other pricing analyses to determine target price range; negotiate and publicize preferential pricing that is applicable to target countries and buyers
Procurement and Supply Management; Demand Visibility	Improve forecasting capabilities to enable procurers to enter longer-term, higher-volume, and/or fixed volume contracts (at country or global level)
Procurement and Supply Management; Pooled Procurement	Establish centralized procurement mechanism to consolidate demand/funding across multiple buyers (including sub-national buyers) to reduce transaction costs/increase leverage
Procurement and Supply Management; Coordinated Supply Planning	Facilitate inter-procurer coordination and data sharing to increase overall market visibility and manage supply security (i.e., ARV procurement working group, coordinated supply planning group, etc.)
Procurement and Supply Management; Variant Optimization	Align key buyers and end users on standardized product packaging, inserts, size, colors, etc. to generate manufacturing efficiencies and cost-savings
Procurement and Supply Management; All-Inclusive Procurement	Expand scope of procurement to include all relevant related products and services (i.e., training, maintenance, etc.) to reduce costs, streamline budgeting, and/or ensure longer-term functionality
Procurement and Supply Management; Product Bundling	Combine procurement of interdependent products from the same or multiple suppliers to reduce prices and maximize patient impact
Procurement and Supply Management; Tender Optimization	Promote best practices in implementing tenders/RfPs (i.e., supplier eligibility, award criteria, timing and duration, reference prices, indicative/minimum volumes, quality assurance, contracting process, etc.)
Introduction and Scale; Forecasting and Quantification	Aggregate on-the-ground data and insights to inform supply planning, procurement, financing, and/or new product introduction strategies
Financial Tools; Prize	Provide financial reward to innovator for achieving a pre-defined R&D outcome
Financial Tools; Development Incentive Grant	Upfront and/or milestone-based payments provided to supplier to pursue agreed upon R&D, regulatory, and/or commercial activities
Financial Tools; Advance Market Commitment	Donors commit to purchasing a minimum volume of products that meet a target product profile (TPP) at an agreed-upon price once developed
Financial Tools; Product Subsidy	Fixed per unit subsidy for predefined period or quantity implemented at any point in distribution chain; includes short-term donations (i.e., catalytic procurement)
Financial Tools; Volume Guarantee	Supplier agrees to lower price in return for sales volume guarantee; guarantor agrees to compensate supplier for any shortfall
Financial Tools; Procurement Guarantee	Guarantee facility provided to intermediate buyer (i.e., procurer) to ensure customer payment will be received on time in full; guarantor assumes risk of default
Financial Tools; Payment Guarantee	Guarantee facility provided to seller (supplier, service provider, etc.) to ensure customer payment will be received on time in full; guarantor assumes risk of default
Financial Tools; Working Capital Facility	Low-cost loans for operating expenditures provided to suppliers, procurers, wholesalers, distributors, etc. with liquidity needs
Financial Tools; Impact Investment	Financing provided to companies that aim to achieve both social impact and financial return in the form of debt, equity, or mixed instruments
Financial Tools; Regulatory Incentive	Rewards for developing products for specific patient populations including priority review vouchers, filing fee waivers, tax credits, etc.

We searched gray literature with Google advance search (https://www.google.com/advanced_search). We first identified a list of key actors involved in market shaping. We then ran an advanced search on all key market shaping organizations using market shaping-related strings adapted from academic database searching to identify PDF files, including major market shaping reports and other published materials (see Supplementary material S1). We manually screened and included up to five relevant documents from each organization (key market shaping organization list and searching results in Supplementary material S2).

### 2.2 Study selection

Search results were imported into Covidence for independent screening by two reviewers. First, the title and abstract were screened and the full-text followed. Excluded articles were recorded with an explanation for exclusion. Any inconsistencies among the reviewers were settled by discussion and resolved with final consensus.

Inclusion criteria are: (1) Population: human; no age limit; in specific LMIC(s), or LMIC(s) is included as part of a global or multiple countries study; (2) Intervention: market shaping activities including but not limited to those listed in Table 1; (3) Outcome: cost; availability/supply; uptake/demand; quality; sustainability; unintended consequence; health outputs; health impact; quality data, analytics and institutional capacity to support effective market performance, strategy planning and execution; innovation; domestic manufacture; (4) Study type: observational/experimental/qualitative/systematic review; for systematic review, references were checked to avoid duplicate and evaluate if any of the cited articles were eligible to be included; and (5) Address at least one of our research questions. LMICs were defined based on the World Bank Income Classification list.

Exclusion criteria are: (1) Does not address any of our research questions; (2) Not a market shaping intervention; (3) National, subnational study; (4) Incorrect study design (clinical trial, cross-sectional survey, etc.); and (5) Not drug, device, diagnostic or vaccine. As mentioned earlier, there is no clear, widely accepted boundary between global market shaping interventions and those at a national level. To operationalize this review, the scope was limited to the global and regional level.

### 2.3 Quality assessment

The Mixed Methods Appraisal Tool (MMAT) Version 2018 was used for quality assessment. The MMAT can be used to appraise the quality of empirical studies, which encompasses the majority of study methodologies included in this review. The MMAT features seven questions that allows the appraisal of most common types of study methodologies and design in a comparable way. Studies are scored out of five based on specific criteria for each study type. A score of 5/5 represents 100% of quality criteria have been met, 4/5 represents 80% of quality criteria have been met, and so on.

### 2.4 Data synthesis and analysis

Included articles were exported from Covidence and two researchers performed data extraction with a structured data extraction template consisting of 7 coding questions and 7 descriptive questions to aid analysis (Table 2).

**Table 2:** Data extraction and analysis.

**#**	**Question**	**Specifications**	**Objectives**	**Analysis**
**Coding questions**				
1	Eligibility	Select from *include* or *exclude*	Obj 1	Descriptive
2	First author affiliated with organizations based in HICs	Select from *yes* or *no*	Obj 1	Descriptive
3	Last or corresponding author affiliated with organizations based in HICs	Select from *yes* or *no*	Obj 1	Descriptive
4	Any author(s) affiliated with organizations in LMICs	Select from *yes* or *no*	Obj 1	Descriptive
5	Any author(s) affiliated with key actors or organizations	Select from *yes* or *no*	Obj 1	Descriptive
6	Product type	Select from *drug, vaccine, device*, and *diagnostic*	Obj 1	Descriptive
7	Geographic location	Select from *Global/LMICs*, WHO regions—*Africa, America, South-East Asia, Europe, Eastern Mediterranean, and Western Pacific*	Obj 1	Descriptive
8	Market shaping category	Select from *R&D, regulatory and guideline, manufacture, procurement, supply, introduction and scale, financing tools*	Obj 1	Descriptive
**Open questions**				
1	Health conditions		Obj 1	Descriptive
2	Products		Obj 1	Descriptive
3	Description of market shaping intervention(s)		Obj 1	Descriptive
4	Investment amount		Obj 1	Descriptive
5	Key actors or organizations		Obj 1	Descriptive
6	Impact	Direct impact—affordability, availability, assured quality, awareness, health outputs; Indirect and other impacts	Obj 2	Deductive and inductive
7	Lessons, enablers and barriers		Obj 3	Inductive

Descriptive analysis was applied to all questions addressing objective 1. For objective 2, we attempted to organize impact into several categories where deductive analysis was performed. However, under each impact category, we performed inductive analysis to capture the emerging themes and patterns from the literature. For objective 3, we performed inductive content analysis which enables the analysis to be driven by the emerging themes and concepts from the literature. When developing the codebook, one experienced researcher developed the first round of the codebook which was then iterated on with the larger research team. A second reviewer then coded the articles, and discussed any challenges with the team. The majority of articles included were qualitative or mixed methods, and missing data was not a concern with this study.

## 3 Results

### 3.1 Description of included articles

**Figure 1:**
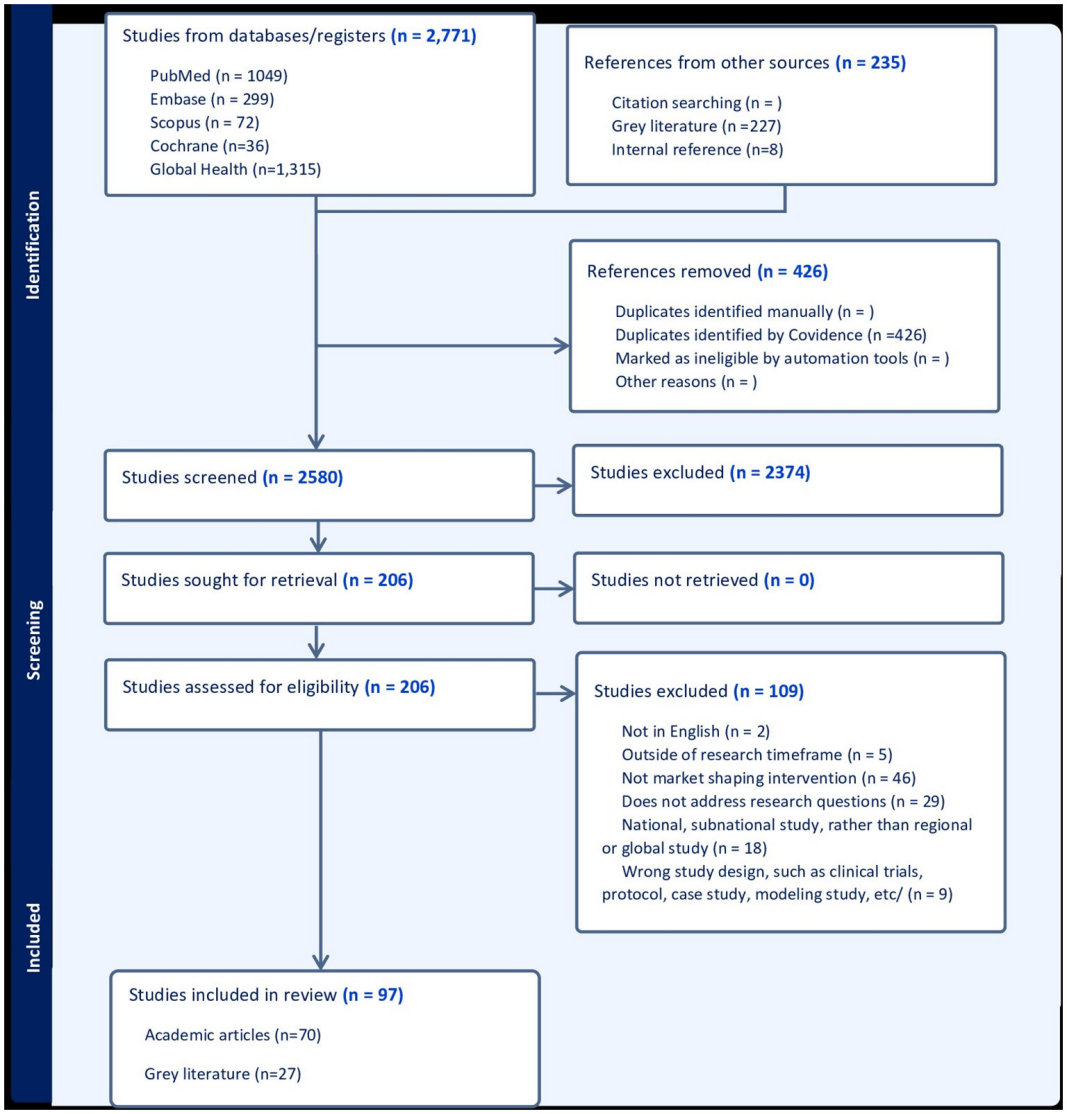
PRISMA flowchart.

The search yielded 3,006 articles and following independent screening, 97 eligible studies were included into the analysis, among which 70 were published articles and 27 were gray literature (Figure 1) (1, 5–100). Of the included studies, there were 62 qualitative studies, 20 quantitative articles, 14 mixed methods studies, and one systematic review. The 70 journal articles were published by 50 different journals, and the most frequently used included five from *Vaccines* and four from *BMJ Global Health*, and four from the *American Journal of Tropical Medicine*. The gray literature documents spanned various organizations including Unitaid, PATH, Gavi and the Clinton Health Access Initiative (CHAI).

Two-thirds of the included articles were published in or after 2018 (Supplementary Figure S1a). A majority of the authors were affiliated with institutions based in high-income countries; only 21 out of 70 (30%) academic articles, and three out of the 27 (11.1%) gray literature documents have authors from LMICs. The key market shaping organizations (see Supplementary material S2 for full list of key organizations) contributed to 14 (20%) academic and 26 (96.3%) gray literature articles. Ninety six articles are assessed with MMAT while MMAT is not able to assess one systematic review. The quality assessment for each article can be found in Supplementary material S4. The 62 qualitative studies averagely scored 4.03 (out of 5). The 20 quantitative articles had an average quality score of 4.80/5, and the average quality scores of the 14 mixed methods studies were 4.54/5. The high quality of included articles helped to guide the descriptive synthesis of results and strengthen the reliability of these findings.

### 3.2 Trends and patterns of market shaping in LMICs in the past decade

Almost half of the market shaping literature focused on drugs (Supplementary Figure S1b). A majority (72/97, 74.2%) of the studies reported market shaping at global level, followed by 16 (16.5%) articles solely from the Africa region, and seven (7.2%) from Southeast Asia (Supplementary Figure S1c). Market shaping interventions addressing manufacturing (33), R&D (23), and procurement (25) were the most reported in articles (Supplementary Figure S1d). Infectious diseases (69/97, 71.1%), were the most reported health conditions receiving market shaping (Supplementary Figure S1e), with malaria, HIV, TB, and neglected tropical diseases (excluding malaria) as the top reported diseases (Supplementary Figure S1f).

We identified the following 11 key market shaping actors or organizations, that have been actively included in the design, implementation, and evaluation of market shaping interventions. These key actors were mentioned in two-thirds of the included articles (64/97, 66%): World Health Organization (WHO), Bill and Melinda Gates Foundation (BMGF), U.S. Food and Drug Administration (US FDA), Gavi, the Vaccine Alliance (Gavi), United Nations Children's Fund (UNICEF), Unitaid, Clinton Health Access Initiative (CHAI), The Drugs for Neglected Diseases initiative (DNDi), PATH, Medicines for Malaria Venture (MMV) and Pan American Health Organization (PAHO).

### 3.3 The impact of market shaping on global health

#### 3.3.1 Impact measurement

The aim of the impact measurement section is to provide an overall assessment and highlight gaps in evaluation and indicators used. Various impact measurements have been used in literature. The most frequently reported impact domains are availability (53/97, 54.6%) and affordability (24/83, 24.7%). There was less focus on quality (9), awareness (7) or uptake/coverage (7). Among all 97 articles, only 15 (15.5%) articles reported three or more impact domains.

However, a variety of measurements are used even under the same impact domain (Table 3). For example, when articles reported “**availability**”-related impact, they measured R&D/regulatory aspects, i.e., new products brought to market and additional products in the R&D pipeline, and the supply and procurement level availabilities, i.e., number of suppliers, quantity of procurement or purchase (32, 72). No retail level availability, such as at pharmacies or the final point of service, has been reported. The most reported indicator for **affordability** was unit price, which was normally compared with the price before market shaping to illustrate the change (7, 54). Only one article reported the full course cost of treatment, and compared the cost across different countries (9). However, no article links price with capacity to pay (such as GDP, salary, etc.).

**Table 3:** Summary of impacts and their measurements reported in the literature.

**Impact domains**	**Measurements and examples**
**Availability**	
**1. R&D**	**(1) Regulatory approval (and time to approval); (2) Access to IP; (3) New drug brought to market; (4) New drugs in pipeline** •*Registered in India (2002) and Germany initially; received orphan drug designation in the EU in 2002 and in the US in 2006; WHO included in the EML in 2011* (28).
**2. Supply and Procurement**	**(1) New suppliers/supply base; (2) Procurement; (3) Supply meet demand; (4) Supply shortage/interruptions; (5) Supply stockpile/rolling buffer** •*The vaccine supply base has grown from 5 manufacturers in 5 countries in 2001 to 15, in 11 countries, in 2016 for DTP vaccines* (37).
**Affordability**	
**1. Price**	**(1) Unit price; (2) Full course price** •…*Advance Market Commitment for pneumococcal conjugate vaccine, a price reduction from $120/dose in the US market to $3.50/dose in the LIC market was achieved* (7).
**2. Indirect price impacts**	**(1) Logistics cost; (2) Price offered by dominant market players creating downward pressure for other suppliers to lower their prices** •*Implementing demand forecasting system with increased storage and transport frequency elevated the variety of efficaciously administered vaccine doses and lowered the logistics cost per dose as much as 34%* (31).
**Assured quality**	
**1. Process to ensure quality**	**(1) Better formulation; (2) Safety statement** •*Moreover, the formulation of Eurartesim means that it has a 2-year shelf life in disease-endemic countries…Following EMA registration, MMV and Sigma-Tau worked together to produce a safety statement on Eurartesim to guide correct prescription of the product* (8).
**2. Products**	**(1) Management on falsified products; (2) Use of non-quality assured products** •*The ensuing enforcement activities resulted in the seizure of thousands of falsified ACTs, which yielded an immediate public health benefit by removing the illicit products from the market place* (19).
**Awareness**	
**Provider/policy maker**	**Awareness of provider/policy maker** •*The stakeholders (doctors, pharmacists, nurses and other service providers at management cadres) were aware of the EDL up to PHC levels,… In Uttarakhand, the EDL is neither available nor displayed in the facilities* (66).
**Uptake/coverage**	
**Population level**	**Proportion of population using the health product** •*Berkley reported that immunization coverage of DTP 2-containing vaccines has increased by 20%-points since the launch of Gavi support for poorest countries* (37).
**Health outputs/outcomes**	
**1. Prevention**	**(1) Case averted; (2) Population protected; (3) Vaccination rates** •*Estimated 14 million additional people have been protected by 3GIRS procured at the NgenIRS negotiated volume discount pricing during the same 3 year period* (72).
**2. Diagnosis**	**(1) Number of test and treat; (2) Number of tests** •*In Brazil to date more than 1,600 P. vivax patients have been tested and treated with single-dose tafenoquine* (84).
**3. Treatment**	**(1) Cure; (2) Lives saved; (3) Mortality rates; (4) Adherence to treatment regimen** •*Since 1995–2000, AIDS mortality fell by nearly 54% in Sao Paulo alone* (40).

For less- reported impact domains, the measurement of impact is less comprehensive. For example, a few articles measure the impact of market shaping on the **awareness** of providers and policy makers but only one article has reported on the awareness of patients or end users (27, 43, 66, 91). In contrast, **service coverage/uptake** was mostly measured at the patient level, while only one article reported policy coverage (37, 85, 86, 100).

Indirect impacts were also reported in various ways, including reduced vaccine wastage as an impact of market shaping for cold chain equipment, south-south technology transfer, and capacity building (10, 23, 36).

#### 3.3.2 Observed impact of different market shaping interventions

Table 4 lists the specific impact metrics of market shaping interventions that were reported across all included articles. The reported impact metrics have been organized based on the type of market shaping intervention, such as those highlighted below.

**Table 4:** Impacts of different market shaping interventions, across all included articles.

**Product/health concern**	**Impact**	**Measurement**	**Description of impacts**
**Product-development partnership (PDP)**			
Fexinidazole (HAT)	Availability	Regulatory approval	*Sanofi has expanded their support to fight Trypanosoma brucei gambiense human African trypanosomiasis (HAT), more commonly known as sleeping sickness, through the development of Fexinidazole. This new oral treatment for early and late stages of the disease received a positive scientific opinion from the European Medicines Agency (EMA) in 2018* (87).
Malaria, human African trypanosomiasis (HAT), Chagas, and visceral leishmaniasis	Availability	New drugs in pipeline	*Since the establishment of MMV in 1999, 17 drug candidates have progressed to preclinical development. Similarly, since its establishment in 2003, DNDi has delivered, recommended, and implemented 7 new treatments against diseases such as malaria (in partnership with Medicines for Malaria Venture), HAT, visceral leishmaniasis, and the world's first pediatric Chagas treatment* (30).
G6PD diagnostic (malaria)	Availability	Regulatory approval	*In 2017, the standard G6PD test was registered in India and Thailand becoming the first quantitative G6PD point of care test available in a malaria endemic country. By 2018, the project led to one commercial product which received SRA in 2021; several other products in development, the approved test was being distributed in 30 countries by 2021* (84).
Coartem dispersible, Eurartesim, Pyramax, artesunate-amodiaquine [ASAQ] and artesunate-mefloquine [ASMQ] (malaria)	Affordability	Full course price	*Only limited information is available about the prices at which they are sold (which may be higher or lower than the product cost) and their affordability within countries. Evidence from Burundi shows that a course of ASAQ is US$0.16 in the public sector, or 40% of a day's wage. However, its price in the private sector is higher (US$0.56), amounting to a day and a half's wages. In the Democratic Republic of Congo and Madagascar, 90% and 92.5%, respectively, of ASAQ treatments are distributed for free. Among outlets that sell ASAQ in the Democratic Republic of Congo, the median price is US$2.72 (roughly 2.4 days' wages) in public facilities and US$3.43 (roughly 3 days' wages) in private facilities. (To determine this, we calculated a rough daily wage derived from the gross domestic product per capita of the Democratic Republic of Congo* [*US$300]. We assumed a 5-day work week* [*although 7 days may be more accurate], dividing 300 by 261, to generate a likely daily wage* [*US$1.15].) In Cambodia, 61.2% of ASMQ treatments are distributed free of cost. Among outlets that sell ASMQ, the median price is US$1.18 (roughly 15% of a day's wage). (We based our calculation on Cambodia's gross domestic product per capita of US$2,100.) In these 4 countries, then, ASAQ and ASMQ are either free or moderately affordable, particularly in the public sector* (9).
Eurartesim tablets (malaria)	Assured quality	Safety statement	*Eurartesim is generally well tolerated and has a simple dosage regimen that involves weight-based administration once daily (up to four tablets per dose) for three days. This makes the drug more patient friendly and could increase compliance over other currently-available forms of ACT requiring twice-daily administration. Owing to the long half-life of piperaquine, Eurartesim also provides better and longer protection from new malaria infections than other forms of ACT, as demonstrated during its clinical development programme…Moreover, the formulation of Eurartesim means that it has a two-year shelf life in disease-endemic countries. A recent study reported Eurartesim to be the most stable form of the six DHA-based formulations investigated…Following EMA registration, MMV and Sigma-Tau worked together to produce a safety statement on Eurartesim to guide correct prescription of the product…To enable the optimal deployment and use of Eurartesim, a repeat-dose study is underway in West Africa (The West African Network for Clinical Trials of Antimalarial Drugs: WANECAM) and a safety and effectiveness study is due to commence in Ghana, Burkina Faso, Mozambique and Tanzania in 2013(INDEPTH Effectiveness and Safety Studies of Antimalarials in Africa:INESS)* (8).
Artesunate-mefloquine	Assured quality	Safety statement	*The fixed dose combination of these has been demonstrated to be efficacious and safe for treating uncomplicated malaria in more than 25,000 patients in Thailand, Myanmar, India, and in a large intervention study in Brazil* (10).
G6PD diagnostic (malaria)	Health output	Number of test and treat	*in 2022 in Cambodia, the test enabled equal access to radical cure of P. vivax malaria for males and females for the first time; in Brazil to date more than 1,600 P. vivax patients have been tested and treated with single-dose tafenoquine* (84).
**Public-private partnership (PPP)**			
Meningitis vaccine (Meningitis vaccine project)	Availability	Regulatory approval	*MVP's work led to approaching a broader group of vaccine manufacturers from LMICs;. Received licensing by Indian regulatory authorities in 2009, WHO prequalification in 2013, and to date ~210 million doses have been manufactured and distributed…was granted an export license for the new PsA-TT conjugate vaccine. WHO prequalification was obtained in June 2010* (18).
Miltefosine (Leishmaniasis)	Availability	Regulatory approval	*Miltefosine was registered in India and Germany initially (2002), received orphan drug designation in the EU in 2002 and in the U.S. in 2006, and was included in the WHO's essential medicines list in 2011* (28).
Vaccines	Availability	Supply meet demand	*BI… distributed about 11.5 million vaccine vials, equivalent to 46 million doses, per year … There was a common perception that the PPP could be described as a success because there were no vaccine shortages around the country and supply security had been assured* (38).
Eurartesim film-coated tablet (*P. falciparum* malaria)	Availability	Regulatory approval	*MMV worked closely with Sigma-Tau to produce the response to the CHMP questions, which necessitated three further clinical trials and 25 new non-clinical studies…in June 2011, the CHMP of the EMA adopted a positive opinion, recommending the granting of marketing authorization in Europe… On 27 October 2011, the European Commission granted full marketing authorization…making it the first ACT to be approved by the EMA for the treatment of uncomplicated P. falciparum malaria* (8).
Ebola vaccine (rVSVΔG-ZEBOV-GP)	Availability	Regulatory approval	*The data from all of the Phase I/II/III studies will form the critical clinical components for the filling package to seek licensure from national regulatory agencies… comprehensive manufacturing data capturing the extensive parallel efforts that were implemented to scale up the manufacturing process of the vaccine and implement a commercial manufacturing facility will be pivotal to support licensure. The rVSVΔG-ZEBOV-GP vaccine received a Priority Medicines (PRIME) designation by the European Medicines Agency and Breakthrough Therapy Designation from the FDA* (35).
PsA-TT conjugate vaccine (meningitis A)	Affordability	Price	…*when MVP, after consulting its advisory bodies, chose to become a “virtual vaccine company” and to develop the new Men A conjugate vaccine on its own. This was a critical decision for the project and, in the end, resulted in a low price for the new PsA-TT conjugate vaccine at $U.S. 0.40 per dose. The low price of the final product greatly facilitated discussions with UNICEF and GAVI and helped ensure that the vaccine would be rapidly used at public health scale* (36).
Ebola vaccine (rVSVΔG-ZEBOV-GP)	Health output	Vaccination rates	…*the vaccine was deployed by WHO, in collaboration with the Congolese Ministry of Health and MSF under expanded access in two separate outbreaks…more than 3,000 people were vaccinated before the outbreak was declared over in July* (35).
PsA-TT conjugate vaccine (meningitis A)	Health output	Vaccination rates	*From December 6 to 15, 2010, more than 95% of 1–29-year-old Burkinabes (10.8 million persons) received the vaccine. The 2011 Burkina Faso surveillance data documented the disappearance of Group A meningococcal disease and carriage studies showed that the organism had disappeared, data consistent with the postvaccination establishment of herd immunity* (36).
**Advance market commitments (AMC)**			
PCV vaccine	Availability	New drug brought to market	*SII's PCV10 came to the market in 2020 which was 5 years later than estimated …Both Pfizer and GSK developed 4-dose presentations during the PCV AMC pilot.*. (85).
COVID-19 vaccines	Availability	Procurement	*Since February 2021, over 41 African countries have received 18 million doses of the COVID-19 vaccine from COVAX* (59).
PCV vaccine	Affordability	Price	*Price decrease from $120/dose to $3.50* (7, 85).
PCV vaccine	Uptake/coverage	Proportion of population using the health product	*PCV AMC pilot achieved the objective of accelerating vaccine uptake… Since the start of the AMC pilot in 2010, 60 of the Gavi-73 countries have introduced PCV. Over half of the Gavi-73 countries introduced PCV in the first 4 years of the AMC pilot. Hib and rota introduction took seven and 8 years from introduction, respectively, to reach this level of uptake* (85).
PCV vaccine	Health output	Lives saved	*Between 2010 and 2030, the PCV AMC pilot is estimated to save between 1.4 million and 2.6 million cumulative lives, and avert 90 million to 175 million cumulative DALYs* (85).
**Volume guarantee**			
COVID-19 RDTs	Availability	*Access to IP; New suppliers*	*Volume guarantee of 120 million AG RDTs. Helped introduce new suppliers to the market and facilitated RDT technology transfer to expand manufacturing capacity in LMICs* (69).
Contraceptive implants	Availability	Procurement	*In the first 2 years of the program, volumes exceeded the specified volume guarantee by providing 3.4 million more implants; 4.7 million implants purchased in 2012 and the annual run-rate is nearly 10 million in 2015* (81).
COVID diagnostics	Affordability	Price	*Price w/volume guarantee: US$5 per unit, with new suppliers decreased to US$2.50, then further decreased to US$1–2 per unit* (69).
Contraceptive implants (Jadelle, Implanon)	Affordability	Price	*Price reduced from $18 to $8.50 (Jadelle); Price reduced from $16.50 to $8.50 (Implanon)* (81).
3GIRS	Affordability	Price	*Price reduction from $23 per unit to average of $16 per unit and trending to $15 or less by 2020* (32).
3GIRS	Health output	Population protected; lives saved; cases averted	*Estimated 14 million additional people have been protected by 3GIRS procured at the NgenIRS negotiated volume discount pricing during the same 3-year period; estimated 61.9 million people protected throughout Africa, between 2.6 and 5.2 million malaria cases averted, and between 7,900 and 15,800 lives saved between 2016 and 2018* (72).
Contraceptive implants	Uptake	Proportion of population using the health product	*Increased uptake in multiple countries, and different population groups* (86).
**Regulatory incentive**			
Neglected tropical diseases	Availability	New drugs in pipeline	*PRV program had a positive and statistically significant impact on the development of drugs for less common tropical diseases, but cannot definitively conclude that an increase in development will lead to an increase in approved therapies. From 2012 to 2016, 85% of the new tropical disease development programs were for diseases which had incidence of at least 10 million people per year* (29).
Moxidectin (Onchocerciasis)	Availability	Regulatory approval	*FDA approved moxidectin for onchocerciasis on June 13, 2018 and awarded MDGH a priority review voucher* (34).
Bedaquiline (TB)	Availability	Regulatory approval	*Bedaquiline has been registered in the more than 50 countries that account for nearly all of the patients with multidrug resistant TB. In 2015, Janssen donated 30,000 courses to countries that qualified for support from the Global Fund, and donated an additional 30,000 courses in 2018* (54). *Beyond the donation program, Janssen sells its 6-month course of treatment for $340 in more than 135 countries and provides a volume discount. The voucher helped Janssen offer more affordable prices more quickly, according to a person with knowledge of the company* (54).
Artemether-lumefantrine (*P. falciparum* malaria)	Affordability	Full course price	*In 2009 the FDA awarded Novartis a priority review voucher for the combination drug artemether-lumefantrine to treat Plasmodium falciparum malaria. Novartis had partnered with the Medicines for Malaria Venture to develop this pediatric formulation…After the drug's approval, Novartis promoted access through partnerships with the Medicines for Malaria Venture and the Affordable Medicines Facility-malaria. Over the course of 12 years the partners have distributed more than 390 million treatments in more than 50 countries at a price as low as US$0.38 per course of treatment* (54).
Miltefosine	Affordability	Price	*Price increase from $54 to $64 from 2002–2008 to $94–$130 in 2009–2014; from 2016 onwards, price is $117–$164* (28).
**Pooled procurement**			
ITNs	Affordability	Price	*Global Fund PPM weighted average prices are slightly higher than those of other procurers, mostly due to differences in specifications (Global Fund PPM prices include a standard set of accessories* [*hooks and strings] that cost $0.8–$1.0)* (80).
ITNs	Assured quality	Management on falsified products	*The analysis of the WHO-PQ-listed ITNs shows that both pyrethroid-only LLINs and pyrethroid PBO ITNs meet the Global Fund target of having four or more quality-assured suppliers* (80).
ARVs	Assured quality	Management on falsified products	*The analysis of the FDA-approved, WHO-PQ-listed, and ERP-approved ARVs shows that all major ARVs meet the Global Fund target of having four or more quality-assured suppliers, with the exception of TLE400—a first-line ARV with a lower dose of Efavirenz compared with TLE600—introduced in 2013, for which only two quality-assured suppliers exist, partly due to recent guideline uncertainty and the resulting uncertainty in need* (80).
Drugs	Affordability	Indirect price impacts	*High-volume drug purchases due to pooled procurement have reduced drug prices by 80 % and 85 % of products are provided by multiple generic manufacturers. The variety of products at different price points offers increased health options for greater segments of the population, as those willing to pay full value for non-generic drugs support the private sector* (16).
Gavi supported vaccines	Availability	Supply and procurement	*10 Gavi vaccine markets exhibit acceptable levels of healthy market dynamics in 2022, a decrease from 11 in 2021 due to a regression in the rotavirus vaccine market but is still in line with Gavi healthy market dynamic targets* (79).
Rotavirus vaccine	Availability	Supply shortage/interruptions	*Significant supply disruptions in seven countries necessitating product switches* (79).
Rotavirus vaccine and yellow fever vaccine	Availability	New drug brought to market	*2 products with improved characteristics (liquid rotavirus vaccine and vial container yellow fever vaccine) are newly offered in 2022, compared with 0 in 2021* (79).
DTP3 vaccine	Coverage	Proportion of population using the health product	*DTP3 coverage rebounding in 2022 to 81% coverage in Gavi57 countries, nearing pre-pandemic coverage level which was 83% in 2019* (79).
DTP coverage	Coverage	Proportion of population using the health product	*Increased coverage…immunization coverage of DTP 2-containing vaccines has increased by 20%-points since the launch of Gavi support for poorest countries* (37).
**Demand forecasting**			
Vaccines	Availability	Supply meet demand	*Recently, Huber et al. and Klemm and McPherson found that because of the better communication between the supply chain members, forecasting accuracy improved and resulted in a less shortage of items* (31).
Vaccines	Affordability	Logistics cost	*Mueller et al. investigated the low-income country's supply chain and found that implementing demand forecasting system with increased storage and transport frequency elevated the variety of efficaciously administered vaccine doses and lowered the logistics cost per dose as much as 34%* (31).
Cold chain equipment	Assured quality	Process to ensure quality	…*The Platform also makes manufacturers accountable for a service bundle including delivery, installation and end-user training to ensure high quality execution. By providing funding for this service bundle Gavi is incentivising the creation of a marketplace for maintenance providers* (23).
**Pricing analysis and negotiation**			
HCV diagnostics	Availability	Supply meet demand	*As of December 2019, ≥787 facilities offered HCV screening and/or treatment services across the seven countries* (48).
HCV diagnostics	Affordability	Unit price	*Since 2014, HCV treatment costs in LMIC have decreased dramatically from >US$100 per diagnostic test and US$750–US$900 per 12-week DAA for innovator products. As of December 2019, prices for diagnostics were as low as <US$1 per RDT and US$8.90–US$15 per VL test (depending on testing platform), and 12-week DAA regimens (typically sofosbuvir + daclatasvir) were as low as US$39 in India (a locally manufactured, quality-controlled product, generic sofosbuvir + daclatasvir) and US$60 in Rwanda (a WHO-prequalified product, generic sofosbuvir + daclatasvir), meaning that in some countries, the total commodity “cost-per-cure” was as low as US$80 (Table 2). Note that this figure is based on the commodity pricing for a single individual and does not incorporate case-finding costs. DAA prices remain high (>US$500) in some countries due to various price mark-ups* (48).
HCV diagnosis and treatment	Health output	Number of test and treat; number cured	*As of December 2019, 5,900+ healthcare workers were trained, 2,209,209 patients were screened, and 120,522 patients-initiated treatment. The cure (SVR12) rate was >90%, including at lower-tier facilities* (48).
TB diagnostics (Gene Xpert MTB/RIF)	Affordability	Price	*IPAQT negotiated with manufacturers (including BD, Cepheid and Hain LifeScience) to obtain concessional pricing (30%−50% discount on existing commercial rates) on equipment and reagents for member laboratories, comparable with price offered to the public sector in high-TB burden low-income countries…The resulting retail prices for WHO-endorsed TB tests were approximately 50% lower for Xpert MTB/RIF (US$67–US$33) and Hain Line Probe Assay (US$58–US$27) and approximately 15% lower for MGIT Liquid Culture (US$18–US$15)….Based on data compiled from non-IPAQT laboratories, we found that IPAQT price of Xpert MTB/RIF ($33.8) was consistently lower than non-IPAQT private market price ($46.7) over the intervention period. Interestingly, non-IPAQT price also experienced a substantial reduction from 2013 to 2017. This suggests that the lower pricing offered by IPAQT laboratories, which are dominant players in the market, may have created a downward pressure on these commercial prices. These prices were also lowest among seven countries with a comparably sized private healthcare sector. Furthermore, the average price in those countries increased from $68.73 in 2015 to $84.53 in 2017 compared with the downward trend observed in India. We could not conduct a similar analysis for other tests as we did not have access to commercial pricing data for those tests from their manufacturers* (44).
TB diagnostics (Gene Xpert MTB/RIF)	Health output	Number of tests	*IPAQT laboratories conducted around 620,000 Xpert MTB/RIF, 47,000 LPAs and 25,000 BacTAlert Liquid Culture tests from 2013 to 2018. For all WHO-endorsed tests combined, testing volume increased from ~28,000 tests in 2013 to ~275,000 tests in 2018…Based on data obtained from Cepheid, we estimated that IPAQT contributed more than 80% of total Xpert volume in the private sector in India* (44).
**Licensing agreements**			
ARVs	Availability	Access to IP	*8 out of 15 SADC countries made use of TRIPS flexibility for the provision of HIV medication amounting to 15 instances total in the region, 4 of the least developed countries (LDC) declared to invoke for the measure of all medicines. 6 of the 15 instances were compulsory licenses or government use, and 9 of the 15 were non-enforcement of patents using the LDC transition provision for pharmaceuticals* (39).
ARVs	Health output	Mortality rate	*Since 1995–2000, AIDs mortality fell by nearly 54% in Sao Paulo alone* (40).
**Streamlining regulatory process, guideline inclusion**			
HIV self-tests (HIVST)	Availability	Procurement	*Consistent engagement with MoH, Global Fund, and PEPFAR of initiative's findings allowed for HIV self-testing to be integrated into longer-term scale-up plans and funding. In 2018, HIV self-testing was included as a dedicated testing strategy in PEPFAR country guidance and received a substantial funding increase* (43).
HIV self-tests	Awareness	Awareness of provider/policy maker	*Increase awareness and willingness to introduce HIV ST…As of July 2018, 59 countries now have policies explicitly allowing HIVST, of which 28 are now fully implementing. In addition, 32 countries are actively piloting HIVST* (43).
**Quality assurance testing**			
Malaria RDTs	Awareness	Awareness of provider/policy maker	*Increase* (27).
Medicines for HIV, TB, malaria	Assured quality	Management on falsified products	*A good example of how GF-JIATF's activities and data collection lead the way to proactive enforcement measures took place in Togo in May 2014. Together with Interpol and national authorities, the operation was directly shaped by GF-JIATF's detailed targeted intelligence. The ensuing enforcement activities resulted in the seizure of thousands of falsified ACTs, which yielded an immediate public health benefit by removing the illicit products from the market place. By deploying on the ground in support of national authorities, GF-JIATF is also able to provide the necessary operational guidance to promote follow-up investigations focused on criminal networks that support the importation and distribution of illicit pharmaceuticals* (19).
**New supplier entry**			
3GIRS	Availability	New drugs in pipeline	*1 product in 2016; in 2019, 3 products in the market with 2 in the pipeline* (72).
Gavi supported vaccines	Availability	New suppliers/supply base	*Gavi has steadily increased the number of vaccine suppliers from 5 in 2001 to 13 in 2010 (for emerging vaccine suppliers)* (7).
DTP vaccines	Availability	New suppliers/supply base	*5 manufacturers in 5 countries (2001) to 15 manufacturers in 11 countries (2016)* (37).

**Product Development and Public-Private Partnerships** have been reported to increase new drugs in the pipeline, facilitate regulatory approval of new health products, reduce prices, and improve health outcomes (Table 4).

**Volume Guarantees** have been observed to increase the number of manufacturers in the market and purchase quantities, and reduce price (Table 4).

**Pooled Procurement** was found to increase the number of vaccine manufacturers, create markets where supply meets demand, reduce price, and increase vaccination coverage (Table 4). Other impacts observed from pooled procurement mechanisms include an increased unit price due to required item specifications, creation of a maintenance marketplace for quality assurance of cold-chain equipment, and meeting a sufficient number of quality-assured suppliers (Table 4).

**Advance Market Commitments (AMC)** increased vaccine doses available to countries and enabled price reductions (Table 4). An external evaluation of the Gavi pneumococcal conjugate vaccine (PCV) AMC found insufficient evidence of accelerating R&D and ineffectiveness at driving price competition, but determined success at driving presentation innovation with the introduction of multi-dose vials, increasing supply due to predictable demand, and increasing vaccine uptake and coverage (Table 4) (85).

**Regulatory Incentives**, such as the priority review voucher, were found to facilitate new drug registrations, new drug approvals, and price reductions, though one article reported a price increase (Table 4) (28).

## 4 Lessons, enablers, and barriers

### 4.1 Key barriers affecting market shaping interventions

Many articles mentioned downstream factors as a barrier to market shaping, including health service delivery (both in public and private sector) and health seeking behavior of target population, as they have often been disconnected from market shaping efforts. Health system capacities must be built to ensure access to health products and services provided through market shaping. One article referenced challenges around country political decisions and capability, “*targets were developed with the assumption that the country had the ability and the capacity to implement the vaccination plan without commensurate support and resources…political considerations often overshadowed the supply chain considerations.”* (92). One article discussed how health staff capacity is a barrier, “*as VL [visceral leishmaniasis] usually clusters in a few and generally remote regions of a country, policy-makers in the capital lack awareness of the disease…Clinical and diagnostic skills are equally concentrated in towns and not freely available in the VL affected areas. Capacity strengthening is jeopardized by the high turnover of health staff, both in clinical duties and in control programs.”* (45). Another article discussed health system barriers at multiple levels that has limited uptake, “*several barriers limit expanded use of GRT in LMICs, including high capital investment and test costs, limited molecular laboratory infrastructure, lack of skilled staff, and need for complex cold-chain sample logistics.”* (22).

Another important downstream factor is the private sector which contributes substantially to the service delivery in many LMICs. One article referenced “*the inclusion of private and public sector facilities in DSF schemes is one mechanism to encourage market competition and improve institutional capacity to deliver drugs…Another consideration is the sustainability of DSF programs reliant on donors for user incentives. Market segmenting can develop the total market by shifting paying customers to the private sector. For example, marketing campaigns could encourage private facility utilization among women, developing the total market and aiding in long term sustainability.”* (16). The private sector also needs to be considered for market shaping as they can be another point where substandard or falsified products enter the market particularly in health systems where end-users often choose between the private and public sectors for care.

Fragmentation and lack of transparency in regulatory processes have also been identified as key barriers for market shaping. Registering products in multiple countries is often time-intensive, and costly for manufacturers to submit registration dossiers to individual countries that may take years to be approved. WHO PQ can be beneficial for improving access to products, but it does not replace regional and national regulatory processes. One article described, “*Despite this progress toward international approvals, manufacturers remain concerned that in some countries the regulatory process remains opaque, the responsible authorities for the registration of HIVST products are still unclear, and even with WHO PQ, in-country validation and registration are still required. These complications add to the cost of doing business for manufacturers and threaten the sustainability of affordable prices for HIVST.”* (43). Harmonization of regulation procedures across countries and more transparency of the process would help decrease the cost of registration requirements.

Additionally, access to intellectual property is another hurdle to market shaping and can impede R&D, innovation, access to certain products, and create monopolies in the market. Investing in and facilitating technology transfer and the licensing of intellectual property, through voluntary licensing or other means, would enable generic manufacturers to enter the market and provide low-cost products to LMICs.

A lack of understanding of commercial partners' incentives and what they view as barriers to market entry was another critical barrier. For market shaping to be successful, competitive markets with multiple product manufacturers would enable sustainable supply and lowered prices. However, introducing new manufacturers may impact financial sustainability amongst the commercial actors due to limited resources. Commercial partners can have challenges prioritizing product candidates for development and products with lower profit margins, which are often the target of market shaping. These products may require additional interventions to be feasible as reported in one article, “*the low-cost design of the FTS was unique to its product line and required its own production run. With competition from other, much higher volume tests for infections such as influenza and HIV, Alere struggled to schedule production of low volume, one-off orders of the FTS, leading to long delays. In addition, the BinaxNOW card test was still available and being used by LF programs. A solution was needed to facilitate the transition to the FTS in country programs and streamline the ordering, production, and shipment process at Alere.”* (67). Market shaping interventions would be more effective through creating sustainable incentives to attract and retain multiple and diverse manufacturers.

### 4.2 Key enablers for effective market shaping

**Figure 2:**
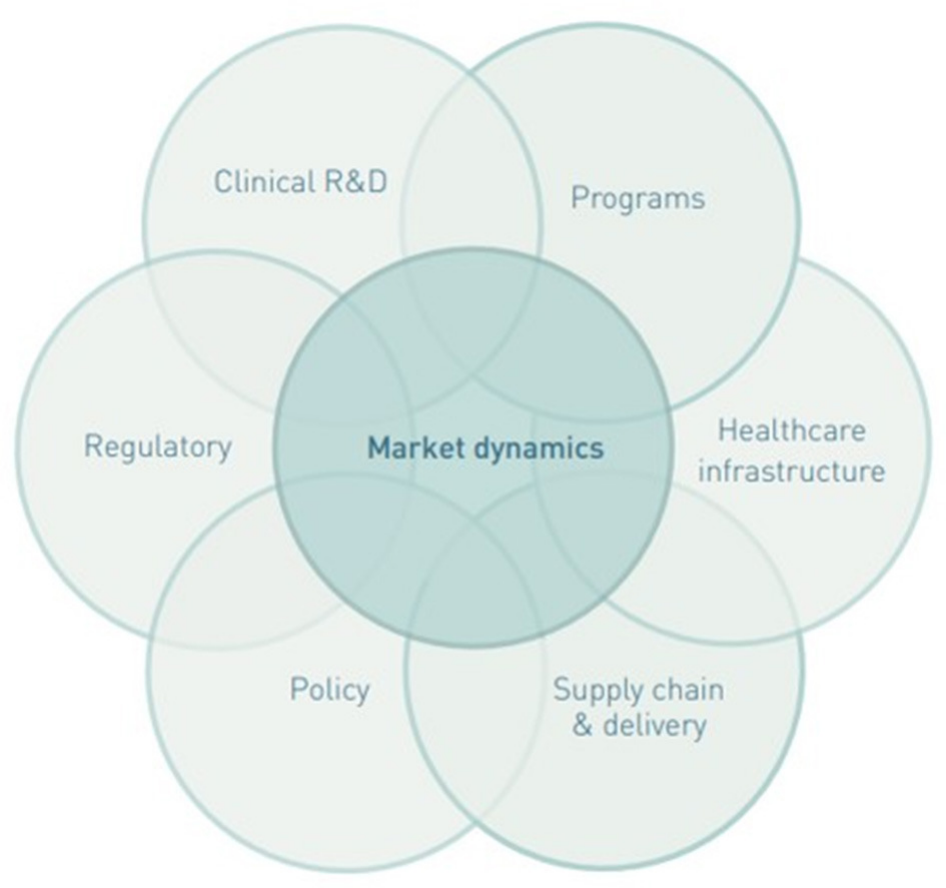
Factors in several overlapping systems that can affect product access (4).

Taking an ecosystem approach to market shaping, by focusing on the continuum across the product and delivery value chain, was an important lesson learned across several articles. A product needs to go through the value chain from R&D, regulatory approval, market entry, procurement, and delivery, to reach the target population (1). The market shaping ecosystem consists of numerous actors from private manufacturers and market shaping organizations to national governments leading to an expanded field of practitioners with overlapping scope along the value chain (Figure 2) (4). To achieve impacts and create a healthy market, actors needed to have vision along the value chain as highlighted in one article, “*wrongfully assumed that the transition would be smooth: just replacing the card test with the FTS in LF programs did not ensure rapid uptake by country LF programs. These programs were not familiar with the new FTS and they were reluctant to change to a diagnostic with different performance characteristics.”* (22). Therefore, additional funding for in-country interventions, such as demand generation activities, may be needed to ensure uptake of health products by the end-user. One article found that “*multifaceted demand generation approaches probably improve adoption, coverage, and sustainability of modern methods used…the success of implementing these strategies include users knowledge about family planning methods, the availability of modern methods, and the accessibility to services.”* (91). For bundled products, like those used in test-and-treat activities such as malaria diagnostics and subsequent treatment, market shaping needs to consider both products to be effective. One article described “*a unique agreement whereby drug-donating companies agreed to support purchase of the diagnostic tests needed to monitor and evaluate the programs they support through their drug donations and other contributions*,” highlighting the importance of understanding bundled products when implementing a market shaping intervention (67).

Within the whole ecosystem, market shaping interventions implemented for some products might affect others due to limited funding and competing priorities. For example, for countries with limited resources, they might choose to provide insulin rather than test strips. Without addressing the overall budget limitation, market shaping interventions on the test strips would not succeed.

Partnerships and coordination across different organizations such as multilateral organizations, government agencies, academic institutions, and private sector actors, are key enablers for all spectrum of market shaping efforts. Coordinating efforts across all partners involved in market shaping can identify market failures where market shaping is needed, mobilize different resources and capacity, limit duplicated efforts and improve efficiency. One article notes, “*Siloed introduction efforts may also contribute to product introduction fatigue among stakeholders at the country level. Moving forward, a key question is how to balance cross-method coordination with method-specific considerations.”* (68). Similarly, at disease or therapeutic area levels, engagement across global, regional, and national stakeholders early in planning can further help with aligning all partners to feasible strategic goals. One article reported, “*this experience demonstrated the value of a public–private partnership for product development of new public health tools with little to no commercial or private market because it was driven by champions from the disease community, the commercial partner, and external donors. The partnership…successfully brought together disease expertise with technical diagnostic development expertise to produce a new monitoring tool which met the performance and field-ready characteristics needed to support LF elimination efforts.”* (67). Regular communication between all partners involved in a market shaping interventions is key for aligning partners on their roles and responsibilities, and addressing challenges as they arise.

Transparent and predictable demand is a pre-condition for many market shaping interventions such as pooled procurement and volume guarantees. By pooling demand from fragmented country markets, demand becomes more predictable and stable. With predictable demand, manufacturers are encouraged to enter markets and limit stockouts, which could help lower prices for commodities and accelerate uptake of new products. One article highlighted a plan to develop two types of demand forecasts for better transparency between market actors, “*Gavi helps improve demand visibility for manufacturers…This increased information transparency is critical for manufacturers to understand growth expectations and desired products to plan production,”* and how both short-term and long-term demand forecasts should be regularly communicated to manufacturers so they can better understand growth expectations to better plan production which is critical to creating a healthy market (23). Another determinant of predictable demand is the capacity of the health system to provide access to the product. One article highlighted “*A further limitation requires that payers be able to accurately predict the number of patients likely to engage in treatment to determine a price point. This requires resources and infrastructure not available in most low-income countries.”* (58).

Longer strategic planning cycles for market shaping activities can enable greater awareness of the time needed to see impact from market shaping interventions, which may be longer than expected. One article touches on the slow progress from approval of a new vaccine to uptake in three countries, “*following phase 3 trial results in 2014, the European Medicines Agency issued a positive scientific opinion for RTS,S malaria vaccine in 2015 and WHO recommended pilot implementation to assess the feasibility of administering four doses of required vaccine in children… It took, however, four years for the world's first malaria vaccine from the demonstration of RTS,S partial protection against malaria in young children before implementation in three sub-Saharan African countries.”* (61).

In cases where market shaping interventions have succeeded, there has been flexible funding or significant contributions from global donors. One article reported on the significant investments by Unitaid, BMGF, and other partners that enabled the price of OraSure to be reduced to US$2 (43). Another article reported on donor funding that extended beyond commodity procurement and allowed for free or heavily subsidized delivery of contraceptive implants (85).

## 5 Discussion

This systematic review aims to describe the evolving pattern and impact of market shaping over the last decade, to identify enablers and barriers to effective market shaping, drawing insights from an extensive collection of published articles and gray literature. However, the majority of articles were published in recent years making it difficult to evaluate larger trends in the field over the last decade. To our best knowledge, this is the only study that synthesis the evidence of a wide range of market shaping interventions on different types of products. Market shaping is an emerging area of interest with various research gaps. Most existing studies generated qualitative evidence, underscoring a pressing need for more quantitative research. Notably, the landscape of market shaping research at global and regional level is currently dominated by actors from high-income countries and a select group of organizations. Many market shaping interventions in the past decade have been driven by donor organization priorities, including priority geographies, which may explain some of the underrepresentation of some global regions. Strikingly, LMICs, the primary target clients and beneficiaries of most market shaping activities, have been largely absent in the market shaping research. This trend reflects broader epistemic imbalances where research in LMICs is often led by global organizations or research institutions in high-income countries. Research on market shaping should be more inclusive, channeling and amplifying voices from LMICs, and explore the impact of market shaping at the national and subnational levels. The included studies disproportionally concentrated on products like drugs and vaccines, predominantly addressing infectious diseases. Other critical areas with different market dynamics call for more evidence such as non-communicable diseases, diagnostic tests, and medical devices, which were underrepresented in the included literature. For example, large upfront investment is needed for medical devices, which is distinct compared to drugs or vaccines. Accordingly, the market shaping for devices might apply different market mechanisms and strategies. Similarly, certain health conditions, such as reproductive and family planning, are intrinsically linked to users' preferences and behavior changes. Addressing market issues in those areas call for additional considerations in the design, implementation and evaluation of market shaping interventions.

The progress of market shaping has been impeded by challenges in impact evaluation and lack of evidence. Currently, impact is not comprehensively or systematically measured, or consistently reported in public domains. Moreover, proxies or intermediary measurements are widely used, while the impact on end-users remains markedly underreported. Existing measurements or approaches are unable to reflect the market dynamics and the evolution of market shaping over the longer term. Herein, it is challenging to attribute outcomes directly to market shaping efforts. Additionally, the majority of included articles were qualitative or descriptive, further limiting the strength of evidence for causal impact. The lack of rigorous quantitative data and causal evaluations in this field should be addressed to enable more critical appraisal of both the positive and unintended impacts of market shaping. A standardized approach to effectively illustrate the value of market shaping is missing, which is much needed to advocate for increased investment and support for such endeavors. A holistic approach should be developed and applied to guide impact evaluation, comprehensively revealing the short-term and long-term impacts of market shaping. Furthermore, in-depth studies examining potential negative impacts and deriving valuable lessons are vital. Market shaping actors are strongly encouraged to enhance knowledge sharing in public domains. Improving the evidence and evaluation could identify and prioritize the most effective and efficient interventions, and potentially unlock more resources for market shaping.

Moving forward, we draw several key recommendations from the existing evidence. First, there is an imperative need to adopt a shared market shaping framework across organizations that implement market shaping interventions. It could serve as foundation and common language for consistent and comparable data and insights, enhance collaborations and partnerships, and align the efforts and strategic goals of diverse stakeholders. Measuring affordability before and after an intervention, and changes in availability of health products could be used as priority benchmarks for evaluation efforts as both of these metrics were reported by a majority of included articles. Standardizing the metrics tracked (e.g., all interventions track unit price before and after) would provide a better opportunity to compare across a variety of market shaping interventions. Addressing enduring bottlenecks to market shaping across product categories and disease areas is crucial, underpinned by access to intellectual property and technology transfer, regulatory support, review and authorization, sustainable incentives for manufacturers, and improvements in demand and supply predictability and coordination. The design and implementation of market shaping initiatives should incorporate end-to-end planning, coordination and capacity building.

Early engagement from market shaping organizations with country ministries of health and diverse local private sector actors could better inform the design and implementation of market shaping interventions. Downstream factors, such as service delivery and health seeking behaviors, are pivotal for access to health products yet unfortunately are too often disconnected from many market shaping interventions. More efforts, including information on service uptake and engagement with health systems and providers, should be placed to market shaping efforts. Finally, creating an enabling environment, characterized by proper time frames and sustainable funding, will be instrumental in ensuring the success and sustainability of market shaping endeavors.

It is important to acknowledge and address several limitations inherent in our study. First, market shaping is a relatively nascent field lacking a clear and consistent definition, established framework, theory, or standardized terminology. Despite our searching strategy involving inputs from a librarian, global health researchers, and market shaping stakeholders, it is likely that some relevant articles may have been missed including those that may have been written in languages other than English. Second, our search of gray literature was limited to up to five articles per organization, and operationalized based on a set of organizations actively involved in market shaping, potentially overlooking emerging, non-traditional, or narrowly specialized actors. Third, our study is susceptible to publication bias, given that nearly all reported impacts of market shaping were positive. Lastly, we acknowledge that published articles and gray literature represent only a fraction of existing market shaping evidence. Potential valuable insights from grant reports, project evaluations, and other non-public domains were not included in this study. Additionally, COVID-19 has likely influenced the field, and at the time of the literature search there were limited evaluation studies examining market shaping's role in pandemic response. Despite these limitations, this study offers valuable evidence shedding light on the practice of market shaping.

## 6 Conclusion

Market shaping interventions have had many positive impacts on access to health products across availability, affordability, awareness, assured quality, and uptake and coverage. In some instances, health outcomes were able to be seen as a result of market shaping interventions such as the estimated lives saved resulting from the pneumococcal vaccine advance market commitment. However, across all market shaping interventions examined in this review, impact was inconsistently measured using a number of metrics such as unit price, new regulatory approval, and number of suppliers among many others which makes it difficult to compare across interventions. Future research should incorporate inputs from authors in low- and middle-income countries and consist of comprehensive program and impact evaluation, cost evaluations, and in-depth studies on the negative impacts of market shaping to better inform the development and implementation of market shaping interventions.

To enhance market shaping in the future, key stakeholders need to adopt shared definitions and frameworks for market shaping to align efforts of various actors; market shaping design and implementation should apply an ecosystem-wide lens, engage with countries and diverse local private sector actors earlier, and put more considerations on service delivery and health seeking behaviors. Key capabilities for market shaping, such as demand forecasting and others, need to be strengthened. Streamlined regulatory processes and stronger enabling environments are needed to ensure the success and sustainability of market shaping endeavors.

## Data Availability

The original contributions presented in the study are included in the article/Supplementary material, further inquiries can be directed to the corresponding author.
