# Supplementary material for: Progress, impacts and lessons from market shaping in the past decade: a systematic review
Source: Front Public Health. 2025 Aug 21;13:1614471. doi: 10.3389/fpubh.2025.1614471 (PMC12408518; doi:10.3389/fpubh.2025.1614471)
Supplement: Supplementary file 1 [file Table_1.docx]

# **S1 Appendix Searching example**

For published articles

| **Group A: Market shaping [title and abstract]** | **Group B: health/products [title and abstract]** | **Group C [title and abstract]** |
| --- | --- | --- |
| **Market shaping[Title/Abstract] OR market dynamic[Title/Abstract] OR priority review voucher[Title/Abstract] OR product development partnership[Title/Abstract] OR supply forecast*[Title/Abstract] OR demand generat*[Title/Abstract] OR demand forecast*[Title/Abstract] OR pooled procure*[Title/Abstract] OR coordinated order[Title/Abstract] OR advance market commitment[Title/Abstract] OR volume guarantee[Title/Abstract] OR channel subsidy[Title/Abstract]** | **vaccine[Title/Abstract] OR diagnos*[Title/Abstract] OR drug[Title/Abstract] OR medicine[Title/Abstract] OR device[Title/Abstract] OR test[Title/Abstract] or contraceptive [title/abstract]** | **(Progress[Title/Abstract] OR pattern[Title/Abstract] OR trend[Title/Abstract] OR change[Title/Abstract] OR evaluat*[Title/Abstract] OR assess*[Title/Abstract] OR observ*[Title/Abstract] OR challenge[Title/Abstract] OR achievement[Title/Abstract] OR lesson[Title/Abstract] OR success[Title/Abstract] OR factor[Title/Abstract] OR enable*[Title/Abstract] OR barrier[Title/Abstract] OR bottleneck[Title/Abstract] OR improve[Title/Abstract] OR gap[Title/Abstract] OR outcome[Title/Abstract] OR impact[Title/Abstract] OR review[Title/Abstract] OR learning [Title/Abstract])** |

For grey literature: "Market shaping" OR "market dynamic" OR "priority review voucher" OR "product development partnership" OR "supply forecast" OR "demand generat" OR "demand forecast" OR "pooled procure" OR "coordinated order" OR "advance market commitment" OR "volume guarantee" OR "channel subsidy"

| **Database** | **Searching equation** |
| --- | --- |
| PubMed  (n=1,049) | ((Market shaping[Title/Abstract] OR market dynamic[Title/Abstract] OR priority review voucher[Title/Abstract] OR product development partnership[Title/Abstract] OR supply forecast*[Title/Abstract] OR demand generat*[Title/Abstract] OR demand forecast*[Title/Abstract] OR pooled procure*[Title/Abstract] OR coordinated order[Title/Abstract] OR advance market commitment[Title/Abstract] OR volume guarantee[Title/Abstract] OR channel subsidy[Title/Abstract]) AND (vaccine[Title/Abstract] OR diagnos*[Title/Abstract] OR drug[Title/Abstract] OR medicine[Title/Abstract] OR device[Title/Abstract] OR test[Title/Abstract] or contraceptive [title/abstract])) AND (Progress[Title/Abstract] OR pattern[Title/Abstract] OR trend[Title/Abstract] OR change[Title/Abstract] OR evaluat*[Title/Abstract] OR assess*[Title/Abstract] OR observ*[Title/Abstract] OR challenge[Title/Abstract] OR achievement[Title/Abstract] OR lesson[Title/Abstract] OR success[Title/Abstract] OR factor[Title/Abstract] OR enable*[Title/Abstract] OR barrier[Title/Abstract] OR bottleneck[Title/Abstract] OR improve[Title/Abstract] OR gap[Title/Abstract] OR outcome[Title/Abstract] OR impact[Title/Abstract] OR review[Title/Abstract] OR learning [Title/Abstract]) |
| Cochrane  (n=36) | Title/ Abstract Market shaping OR market dynamic OR priority review voucher OR product development partnership OR supply forecast* OR demand generat* OR demand forecast* OR pooled procure* OR coordinated order OR advance market commitment OR volume guarantee OR channel subsidy  And Title/ Abstract vaccine OR diagnos* OR drug OR medicine OR device OR test OR contraceptive  And Title/ Abstract Progress OR pattern OR trend OR change OR evaluat* OR assess* OR observ* OR challenge OR achievement OR lesson OR success OR factor OR enable* OR barrier OR bottleneck OR improve OR gap OR outcome OR impact OR review OR learning |
| Embase  (n=299) | Title/ Abstract Market shaping OR market dynamic OR priority review voucher OR product development partnership OR supply forecast* OR demand generat* OR demand forecast* OR pooled procure* OR coordinated order OR advance market commitment OR volume guarantee OR channel subsidy  And Title/ Abstract vaccine OR diagnos* OR drug OR medicine OR device OR test OR contraceptive  And Title/ Abstract Progress OR pattern OR trend OR change OR evaluat* OR assess* OR observ* OR challenge OR achievement OR lesson OR success OR factor OR enable* OR barrier OR bottleneck OR improve OR gap OR outcome OR impact OR review OR learning |
| Scopus  (n=72) | Advanced Search  (market AND shaping OR market AND dynamic OR priority AND review AND voucher OR product AND development AND partnership OR supply AND forecast* OR demand AND generat* OR demand AND forecast* OR pooled AND procure* OR coordinated AND order OR advance AND market AND commitment OR volume AND guarantee OR channel AND subsidy ) AND ( vaccine OR diagnos* OR drug OR medicine OR device OR test OR contraceptive ) AND ( progress OR pattern OR trend OR change OR evaluat* OR assess* OR observ* OR challenge OR achievement OR lesson OR success OR factor OR enable* OR barrier OR bottleneck OR improve OR gap OR outcome OR impact OR review OR learning) |
| Global Health  (n=1,315) | Abstract: Market shaping OR market dynamic OR priority review voucher OR product development partnership OR supply forecast* OR demand generat* OR demand forecast* OR pooled procure* OR coordinated order OR advance market commitment OR volume guarantee OR channel subsidy  AND  Abstract: vaccine OR diagnos* OR drug OR medicine OR device OR test OR contraceptive  AND  Abstract: Progress OR pattern OR trend OR change OR evaluat* OR assess* OR observ* OR challenge OR achievement OR lesson OR success OR factor OR enable* OR barrier OR bottleneck OR improve OR gap OR outcome OR impact OR review OR learning |
