# Supplementary material for: Progress, impacts and lessons from market shaping in the past decade: a systematic review
Source: Front Public Health. 2025 Aug 21;13:1614471. doi: 10.3389/fpubh.2025.1614471 (PMC12408518; doi:10.3389/fpubh.2025.1614471)
Supplement: Supplementary file 2 [file Table_2.docx]

# **S2 Appendix Key market shaping organization and searching results**

|  | ***Original search*** | | | ***Updated search*** | | |
| --- | --- | --- | --- | --- | --- | --- |
|  | **# Search Results (all in title)** | **# Search Results (outside of title)** | **# report to be screened** | **# Search Results (all in title)** | **# Search Results (outside of title)** | **# report to be screened** |
| Access to Medicine Foundation | 0 | 31 | 5 | 0 | 8 | 5 |
| Medicines Patent Pool | 0 | 44 | 5 | 5 | 76 | 5 |
| Innovative Vector Control Consortium | 0 | 44 | 5 | 3 | 4 | 5 |
| SEMA | 0 | 7 | 5 | 2 | 8 | 5 |
| PAHO | 6 | 32 | 9 | 4 | 172 | 5 |
| MedAccess | 1 | 1 | 1 | 1 | 1 (same as all in title) | 1 |
| GARDP | 0 | 1 | 1 | 0 | 57 | 5 |
| IFPMA | 1 | 10 | 5 | 2 | 85 | 5 |
| African Medical Device Forum | 0 | 4 | 4 | 0 | 0 | 0 |
| Medicines for Malaria Venture | 7 | 250 | 5 | 1 | 33 | 5 |
| TB Alliance | 1 | 42 | 6 | 0 | 3 | 3 |
| FP2020 | 0 | 7 | 5 | 0 | 0 | 0 |
| Reproductive Health Supplies Coalition | 33 | 169 | 5 | 8 | 98 | 5 |
| World Bank | 466 |  | 5 | 0 | 0 | 0 |
| Global Financing Facility | 0 | 32 | 5 | 1 | 57 | 5 |
| WHO | 2570 |  | 5 | 2 | 425 | 5 |
| USAID | 374 |  | 5 | 148 |  | 5 |
| UNITAID | 7 |  | 5 | 2 | 40 | 5 |
| UNICEF | 89 | 316 | 10 | 172 |  | 5 |
| R4D | 2 | 29 | 5 | 0 | 22 | 5 |
| PATH | 4 | 91 | 5 | 0 | 0 | 0 |
| Global Fund | 30 |  | 5 | 0 | 0 | 0 |
| Gavi | 2 | 7 | 5 | 0 | 0 | 0 |
| FIND | 2 | 8 | 8 | 6 | 43 | 5 |
| CHAI | 0 | 30 | 5 | 4 | 40 | 5 |
| BMGF | 3 | 22 | 8 | 0 | 2 | 1 (second link is the same as first just in French) |
|  |  |  |  |  |  |  |
| UK FCDO | 0 | 0 |  | 0 | 0 | 0 |
| African Medical Supply Platform | 0 | 0 |  | 0 | 0 | 0 |
| Global Health Investment Fund | 0 | 0 |  | 0 | 0 | 0 |
|  |  |  |  |  |  |  |
| **Total** |  |  | **137** |  |  | **90** |
