# Supplementary material for: Progress, impacts and lessons from market shaping in the past decade: a systematic review
Source: Front Public Health. 2025 Aug 21;13:1614471. doi: 10.3389/fpubh.2025.1614471 (PMC12408518; doi:10.3389/fpubh.2025.1614471)
Supplement: Supplementary file 4 [file Table_4.docx]

# **S4 Appendix Included articles and quality assessment**

| **Ref**  **#** | **Title** | **Authors** | **Year of publication** | **Study design*** | **MMAT score**** | **Primary Reviewer** | **Secondary Reviewer** | **Date of Extraction** |
| --- | --- | --- | --- | --- | --- | --- | --- | --- |
| 1 | An overview of the antiretroviral market. | Camponeschi G et al | 2013 | Mixed | 5 | RC | KO | June-July 2023 |
| 2 | Lessons learned in shaping vaccine markets in low-income countries: a review of the vaccine market segment supported by the GAVI Alliance. | Gilchrist SA & Nanni A | 2013 | Quali | 5 | RC | KO | June-July 2023 |
| 3 | MMV in partnership: the Eurartesimexperience. | Ubben D &Poll EM | 2013 | Quali | 4 | RC | KO | June-July 2023 |
| 4 | Linking research to global health equity: the contribution of product development partnerships to access to medicines and research capacity building. | Pratt B & Loff B | 2013 | Mixed | 5 | RC | KO | June-July 2023 |
| 5 | The story of artesunate-mefloquine (ASMQ), innovative partnerships in drug development: case study. | Wells S et al | 2013 | Quali | 4 | RC | KO | June-July 2023 |
| 6 | Antiretrovirals for low income countries: an analysis of the commercial viability of a highly competitive market. | Nakakeeto ON & Elliott BV | 2013 | Mixed | 5 | RC | KO | Jue-July 2023 |
| 7 | The use of the United States FDA programs as a strategy to advance the development of drug products for neglected tropical diseases. | Sachs-Barrable K et al | 2014 | Quali | 5 | RC | KO | June-July 2023 |
| 8 | Demand generation activities and modern contraceptive use in urban areas of four countries: a longitudinal evaluation. | Speizer IS et al | 2014 | Quant | 5 | RC | KO | June-July 2023 |
| 9 | Impact of BRICS' investment in vaccine development on the global vaccine market. | Kaddar M et al | 2014 | Quali | 5 | RC | KO | June-July 2023 |
| 10 | Experience with the priority review voucher program for drug development. | Kesselheim AS et al | 2015 | Quali | 4 | RC | KO | June-July 2023 |
| 11 | New dialogue for the way forward in maternal health: addressing market inefficiencies. | McCarthy K et al | 2015 | Quali | 4 | RC | KO | June-July 2023 |
| 12 | Pediatric tuberculosis drug market: An insider perspective on challenges and solutions. | Usherenko I et al | 2015 | Quali | 5 | RC | KO | June-July 2023 |
| 13 | From epidemic meningitis vaccines for Africa to the meningitis vaccine project. | Aguado MT et al | 2015 | Quali | 4 | RC | KO | June-July 2023 |
| 14 | The Joint Interagency Task Force and the global steering committee for the quality assurance of health products: two new and proactive approaches promoting access to safe and effective medicines. | Cinnamond M & Woods T. | 2015 | Quali | 1 | RC | KO | June-July 2023 |
| 15 | A review of innovative international financing mechanisms to address noncommunicable diseases. | Meghani A & Basu S. | 2015 | Quali | 5 | RC | KO | June-July 2023 |
| 16 | Fostering incentives for research, development, and delivery of interventions for neglected tropical diseases: lessons from malaria. | Berdud M et al | 2016 | Quali | 4 | RC | KO | June-July 2023 |
| 17 | Affordable HIV drug-resistance testing for monitoring of antiretroviral therapy in sub-Saharan Africa. | Inzaule SC et al | 2016 | Quali | 4 | RC | KO | June-July 2023 |
| 18 | Seizing market shaping opportunities for vaccine cold chain equipment. | Azimi T et al | 2017 | Quali | 5 | RC | KO | June-July 2023 |
| 19 | Priorities for the Priority Review Voucher. | Ridley DB | 2017 | Quali | 5 | RC | KO | June-July 2023 |
| 20 | The tropical disease priority review voucher: a game-changer for tropical disease products. | Berman J & Radhakrishna T | 2017 | Quali | 4 | RC | KO | June-July 2023 |
| 21 | Association of the priority review voucher with neglected tropical disease drug and vaccine development. | Jain N et al | 2017 | Quant | 5 | RC | KO | June-July 2023 |
| 22 | Global survey of malaria rapid diagnostic test (RDT) sales, procurement and lot verification practices: assessing the use of the WHO-FIND Malaria RDT Evaluation Programme (2011-2014). | Incardona S et al | 2017 | Quant | 5 | RC | KO | June-July 2023 |
| 23 | Why miltefosine-a life-saving drug for leishmaniasis-is unavailable to people who need it the most. | Sunyoto T et al | 2018 | Quali | 4 | KO | WM | June-July 2023 |
| 24 | Is the priority review voucher program stimulating new drug development for tropical diseases? | Kerr KW et al | 2018 | Quant | 5 | KO | WM | June-July 2023 |
| 25 | Working towards new drugs against parasitic worms in a public-development partnership. | Preston S & Gasser RB | 2018 | Quali | 5 | KO | WM | June-July 2023 |
| 26 | A fuzzy MICMAC analysis for improving supply chain performance of basic vaccines in developing countries. | Chandra D & Kumar D | 2018 | Quali | 5 | KO | WM | June-July 2023 |
| 27 | Bacillus Calmette-Guérin (BCG) vaccine: A global assessment of demand and supply balance. | Cernuschi T et al | 2018 | Mixed | 4 | KO | WM | June-July 2023 |
| 28 | Buying and selling prioritized regulatory review: The market for priority review vouchers as quasi-intellectual property. | Wang O | 2018 | Quali | 4 | KO | WM | June-July 2023 |
| 29 | Creative use of the priority review voucher by public and not-for-profit actors delivers the first new FDA-approved treatment for river blindness in 20 years. | Olliaro PL et al | 2018 | Quali | 4 | KO | WM | June-July 2023 |
| 30 | Unprecedented pace and partnerships: the story of and lessons learned from one Ebola vaccine program. | Gupta SB et al | 2018 | Quali | 5 | KO | WM | June-July 2023 |
| 31 | Lessons from the Meningitis Vaccine Project. | LaForce FM et al | 2018 | Quali | 5 | KO | WM | June-July 2023 |
| 32 | Vaccines, inspiring innovation in health. | Pagliusi S et al. | 2018 | Quali | 2 | KO | WM | June-July 2023 |
| 33 | An evaluation of South Africa's public-private partnership for the localisation of vaccine research, manufacture and distribution. | Walwyn DR & Nkolele AT | 2018 | Mixed | 5 | KO | WM | June-July 2023 |
| 34 | Patent challenges in the procurement and supply of generic new essential medicines and lessons from HIV in the southern African Development Community (SADC) region. | 't Hoen EFM et al | 2018 | Quali | 5 | KO | WM | June-July 2023 |
| 35 | Affordability versus innovation: Is compulsory licensing the solution? | Pandey E & Paul SB | 2019 | Quali | 5 | VH | KO | June-July 2023 |
| 36 | Regulatory trends in drug development in Asia Pacific. | Han FV & Weiss K | 2019 | Quali | 4 | VH | KO | June-July 2023 |
| 37 | "Running the Gauntlet": Formidable challenges in advancing neglected tropical diseases vaccines from development through licensure, and a "Call to Action". | Bottazzi ME & Hotez PJ | 2019 | Quali | 5 | VH | KO | June-July 2023 |
| 38 | The Self-Testing Africa (STAR) Initiative: accelerating global access and scale-up of HIV self-testing. | Ingold H et al | 2019 | Quali | 5 | VH | KO | June-July 2023 |
| 39 | Initiative for Promoting Affordable and Quality Tuberculosis Tests (IPAQT): a market-shaping intervention in India. | Dabas H et al | 2019 | Quali | 5 | VH | KO | June-July 2023 |
| 40 | Exploring global and country-level barriers to an effective supply of leishmaniasis medicines and diagnostics in eastern Africa: a qualitative study. | Sunyoto T et al | 2019 | Quali | 5 | VH | KO | June-July 2023 |
| 41 | Blood glucose meters and test strips: global market and challenges to access in low-resource settings. | Klatman EL et al | 2019 | Quali | 5 | VH | KO | June-July 2023 |
| 42 | Vaccine procurement in the Middle East and North Africa region: challenges and ways of improving program efficiency and fiscal space. | Kaddar M et al | 2019 | Mixed | 5 | VH | KO | June-July 2023 |
| 43 | Initial success from a public health approach to hepatitis C testing, treatment and cure in seven countries: the road to elimination. | Boeke CE et al | 2020 | Quant | 4 | VH | KO | June-July 2023 |
| 44 | Lessons from one year experience of pooled procurement of pharmaceuticals: exploration of indicators and assessing pharmacies’ performance. | Bastani P et al | 2020 | Mixed | 5 | VH | KO | June-July 2023 |
| 45 | Increasing private sector investment in neglected tropical disease (NTD) research and development: A mixed methods study. | Barofsky J et al | 2020 | Mixed | 2 | VH | KO | June-July 2023 |
| 46 | COVID-19 vaccines for all? | Usher AD | 2020 | Quali | 4 | VH | KO | June-July 2023 |
| 47 | Establishing the value and strategies for respiratory syncytial virus (RSV) control: the European RSV consortium (RESCEU). | Miller M | 2020 | Quali | 1 | VH | KO | June-July 2023 |
| 48 | A situation analysis of the state of supply of in vitro diagnostics in low-income countries. | Stevenson M | 2020 | Quali | 5 | VH | KO | June-July 2023 |
| 49 | Tropical disease priority review vouchers: lessons in promoting drug development and access. | Ridley DB et al | 2021 | Quali | 4 | VH | KO | June-July 2023 |
| 50 | The global procurement landscape of leishmaniasis medicines. | Choi HL et al | 2021 | Quant | 5 | VH | KO | June-July 2023 |
| 51 | European collaborations on medicine and vaccine procurement. | Vogler S et al | 2021 | Quali | 5 | VH | KO | June-July 2023 |
| 52 | Shortage of essential antimicrobials: a major challenge to global health security. | Nusrat S et al | 2021 | Quali | 5 | VH | KO | June-July 2023 |
| 53 | The 'Netflix plus model': can subscription financing improve access to medicines in low- and middle-income countries? | Cherla A et al | 2021 | Quali | 5 | VH | KO | June-July 2023 |
| 54 | Considerations for establishing successful coronavirus disease vaccination programs in Africa. | Williams V et al | 2021 | Quali | 5 | VH | KO | June-July 2023 |
| 55 | A financial and global demand analysis to inform decisions for funding and clinical development of group B Streptococcus vaccines for pregnant women. | Malvolti S et al | 2021 | Quant | 5 | VH | KO | June-July 2023 |
| 56 | Supply and delivery of vaccines for global health. | Excler JL et al | 2021 | Quali | 5 | VH | KO | June-July 2023 |
| 57 | Fexinidazole for human African trypanosomiasis, the fruit of a successful public-private partnership. | Bernhard S et al | 2022 | Quali | 5 | VH | KO | June-July 2023 |
| 58 | Assessing perceptions of establishing a vaccine pooled procurement mechanism for the Western Pacific Region. | Abou-Nader A et al | 2022 | Quali | 4 | VH | KO | June-July 2023 |
| 59 | Global access to existing and future antimicrobials and diagnostics: antimicrobial subscription and pooled procurement. | Berman D et al | 2022 | Quant | 5 | VH | KO | June-July 2023 |
| 60 | Sustainable vaccine manufacturing in low- and middle-income countries. | Hayman B et al | 2022 | Quant | 4 | VH | KO | June-July 2023 |
| 61 | Effectiveness of supply chain planning in ensuring availability of CD/NCD drugs in non-metropolitan and rural public health system. | Sanjib S et al | 2022 | Mixed | 5 | VH | KO | June-July 2023 |
| 62 | Development and introduction of the filariasis test strip: a new diagnostic test for the global program to eliminate lymphatic filariasis. | Pantelias, A et al | 2022 | Quali | 5 | VH | KO | June-July 2023 |
| 63 | What have we learned? Implementation of a shared learning agenda and access strategy for the hormonal intrauterine device. | Rademacher KH et al | 2022 | Mixed | 4 | VH | KO | June-July 2023 |
| 64 | Democratizing access to testing. | FIND | 2022 | Quali | 1 | KO | WM | June-July 2023 |
| 65 | Forecasted demand for current and new ARV medicines in low and middle income countries, 2015-2025. | Medicines Patent Pool, World Health Organization | 2016 | Quant | 5 | KO | WM | June-July 2023 |
| 66 | Projected savings through public health voluntary licences of HIV drugs negotiated by the Medicines Patent Pool (MPP). | Juneja S et al | 2017 | Quant | 5 | KO | WM | June-July 2023 |
| 67 | Market Access Initiatives: NgenIRS and the New Nets Project. | Christen F (IVCC) | 2019 | Quali | 5 | KO | WM | June-July 2023 |
| 68 | Market shaping for family planning: an analysis of current activities and future opportunities to improve the effectiveness of family planning markets. | Dalberg Global Development Advisors/RHSC | 2014 | Quali | 5 | KO | WM | June-July 2023 |
| 69 | Health markets for global health: a market shaping primer. | USAID | 2014 | Quali | 5 | KO | WM | June-July 2023 |
| 70 | Global malaria diagnostic and artemisinin treatment commodities demand forecast 2016-2019. | WHO/Unitaid | 2016 | Quant | 5 | KO | WM | June-July 2023 |
| 71 | Global malaria diagnostic and artemisinin treatment commodities demand forecast 2017-2020. | WHO/Unitaid | 2017 | Quant | 5 | KO | WM | June-July 2023 |
| 72 | Global malaria diagnostic and artemisinin treatment commodities demand forecast 2017-2021. | WHO/Unitaid | 2018 | Quant | 5 | KO | WM | June-July 2023 |
| 73 | Global malaria diagnostic and artemisinin treatment commodities demand forecast 2015-2018. | WHO/Unitaid | 2016 | Quant | 5 | KO | WM | June-July 2023 |
| 74 | ACT demand forecast, 2012-2013. | Unitaid | 2012 | Quant | 5 | KO | WM | June-July 2023 |
| 75 | Gavi, the Vaccine Alliance: strategic update. | Baker | 2023 | Quant | 3 | KO | WM | June-July 2023 |
| 76 | Technical evaluation reference group: market shaping strategy mid-term review position paper. | Global Fund | 2019 | Mixed | 5 | KO | WM | June-July 2023 |
| 77 | Case study: expanding global access to contraceptive implants. | CHAI | 2015 | Quali | 5 | KO | WM | June-July 2023 |
| 78 | Annual report 2021. | CHAI | 2021 | Quali | 1 | KO | WM | June-July 2023 |
| 79 | How portfolio-based product development can accelerate progress in global health. | PATH | 2021 | Quali | 1 | KO | WM | June-July 2023 |
| 80 | G6PD diagnostics: improving health outcomes and breaking the cycle of malaria transmission. | PATH | 2022 | Quali | 1 | KO | WM | June-July 2023 |
| 81 | Gavi PCV AMC Pilot: 2nd outcomes and impact evaluation. | Gavi/Dalberg | 2021 | Mixed | 5 | KO | WM | June-July 2023 |
| 82 | Liftoff: the blossoming of contraceptive implant use in Africa. | Jacobstein | 2018 | Quant | 5 | KO | WM | June-July 2023 |
| 83 | Collaborating to end neglected tropical diseases: catalyzing innovation and partnerships. | IFPMA | 2020 | Quali | 1 | KO | WM | June-July 2023 |
| 84 | Alternative funding models for medical innovation: the role of product development partnerships in product innovation for infectious diseases | Kourouklis D et al | 2022 | Quant | 5 | KO | WM | June-July 2023 |
| 85 | Does structural form matter? A comparative analysis of pooled procurement mechanisms for health commodities | Parmaksiz K et al | 2023 | Quali | 5 | KO | WM | June-July 2024 |
| 86 | The role of global health partnerships in vaccine equity: a scoping review | Nunes C et al | 2023 | Quali | 5 | KO | WM | June-July 2024 |
| 87 | Implementation strategies, facilitators, and barriers to scaling up and sustaining demand generation in family planning, a mixed-methods systematic review | Nabhan A et al | 2023 | SR | NA | KO | WM | June-July 2024 |
| 88 | COVID-19 Vaccine Collaborative Supply Planning: Is This the Next Frontier for Routine Immunization Supply Chains | Akhlaghi L et al | 2024 | Mixed | 4 | KO | WM | June-July 2024 |
| 90 | From promise to practice: a guide to developing pooled procurement mechanisms for medicines and vaccines | Parmaksiz K et al | 2023 | Quali | 5 | KO | WM | June-July 2024 |
| 91 | Analysis of the first ten years of FDA’s rare pediatric disease priority review voucher program: designations, diseases, and drug development | Mease C et al | 2024 | Quant | 5 | KO | WM | June-July 2024 |
| 92 | Transformative partnership between the Medicines Patent Pool and ViiV Healthcare enables 24 million people in low- and middle-income countries to access innovative HIV treatment | Medicines Patent Pool | 2024 | Quali | 1 | KO | WM | June-July 2024 |
| 93 | IVCC Annual Report 2022-2023 | IVCC | 2023 | Quali | 1 | KO | WM | June-July 2024 |
| 94 | Update on the rollout of COVID-19 tools | ACT-A Tracking & Monitoring Task Force | 2023 | Quali | 3 | KO | WM | June-July 2024 |
| 95 | Discerning Demand: A Guide to Scale-Driven Product Development and Introduction | Nepomnyashchiy L et al | 2023 | Quali | 5 | KO | WM | June-July 2024 |
| 96 | Evaluation of Unitaid’s Antiretroviral Therapy (ART) Optimisation Portfolio | Sequeira FP et al | 2022 | Mixed | 5 | KO | WM | June-July 2024 |
| 97 | Maternal & Child Wasting Products | Harris D & R4D | 2024 | Quali | 4 | KO | WM | June-July 2024 |
| 98 | CHAI Market Shaping Framework | CHAI | 2024 | Quali | 2 | KO | WM | June-July 2024 |

*Mixed= mixed methods; Quali=qualitative; Quant=quantitative; SR = systematic review

**ranges from 0-5 while 5 indicates the highest quality
