# Supplementary figures and images for: Progress, impacts and lessons from market shaping in the past decade: a systematic review
Source: Front Public Health. 2025 Aug 21;13:1614471. doi: 10.3389/fpubh.2025.1614471 (PMC12408518; doi:10.3389/fpubh.2025.1614471)

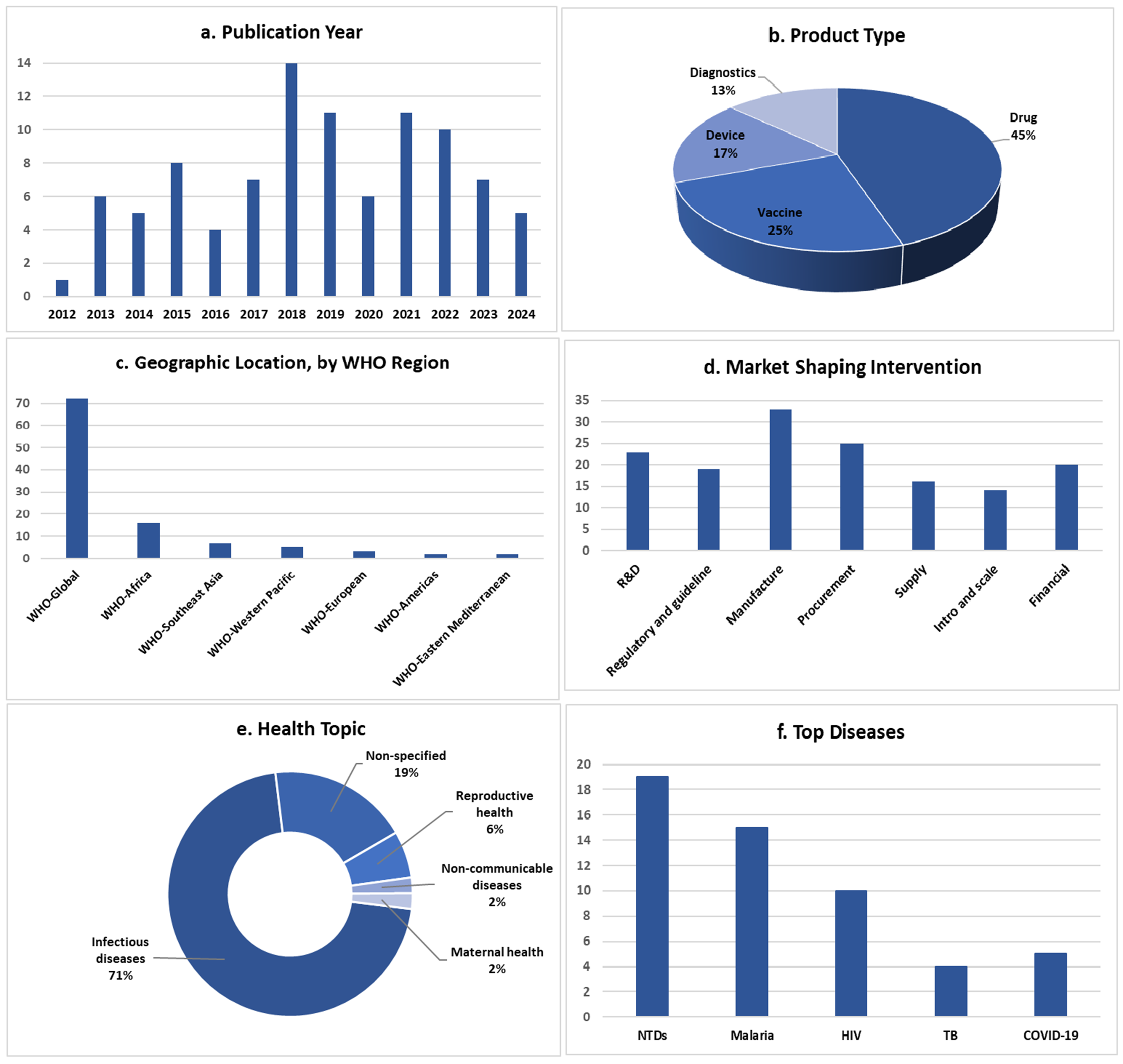

Supplement: Supplementary file 5 [file Image_1.tif]

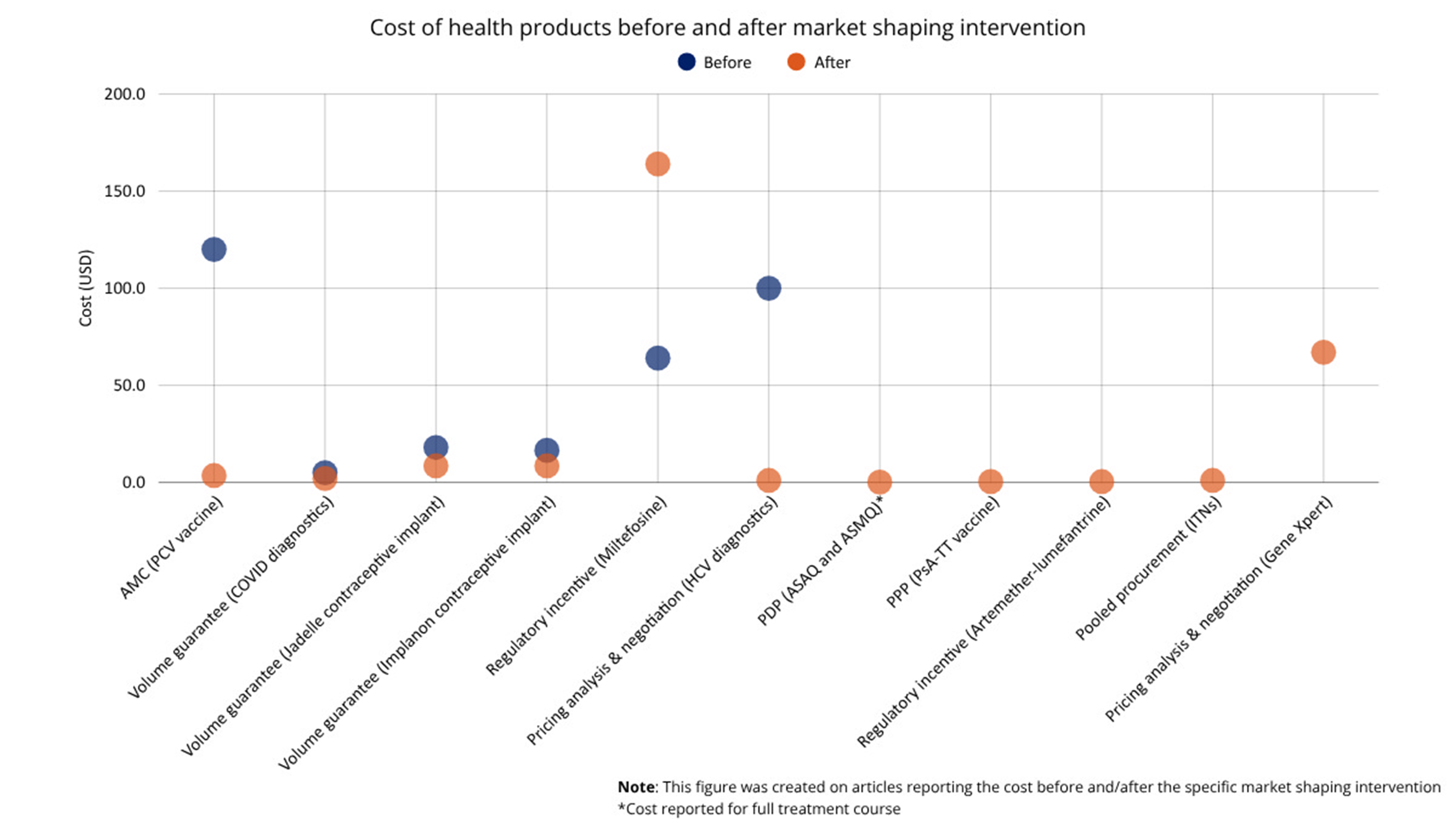

Supplement: Supplementary file 6 [file Image_2.tiff]
